# Sex differences in preterm cytokine and inflammasome responses and modulation by exogenous sex steroids

**DOI:** 10.1038/s41390-025-04350-0

**Published:** 2025-09-24

**Authors:** Matthew McGovern, Lynne A. Kelly, Rebecca Finnegan, John F. Murphy, John Kelleher, Ashanty M. Melo, Catherine M. Greene, Eleanor J. Molloy

**Affiliations:** 1 Discipline of Paediatrics, Trinity College, the University of Dublin, Dublin, Ireland; 2 Trinity Translational Medicine Institute (TTMI) & Trinity Research in Childhood Centre (TRICC), Trinity College Dublin, Dublin, Ireland; 3 Department of Clinical Microbiology, The Royal College of Surgeons in Ireland, Dublin, Ireland; 4 Department of Neonatology, National Maternity Hospital, Holles Street, Dublin, Ireland; 5 Neonatology, Coombe Hospital, Dublin, Ireland; 6 Neurodisability, Children’s Health Ireland (CHI) at Tallaght, Dublin, Ireland; 7 Neonatology, CHI at Crumlin, Dublin, Ireland

## Abstract

**Background:**

Preterm infants are at increased risk of sepsis compared to adults and older children. Preterm immune cells have altered cytokine responses compared to term neonates and adults and all have sex-related differences in immunity. We examined inflammasome activation and cytokines with endotoxin and sex steroid hormones between preterm and term neonates.

**Methods:**

Preterm (*n* = 40) and term (*n* = 32) peripheral blood samples were incubated with Lipopolysaccharide (LPS), Estradiol (E2), Progesterone (Pg) or Pam3CSK4 and biomarkers were analysed by ELISA. Inflammasome genes, NLR family pyrin domain containing 3 (NLRP3), apoptosis-associated speck-like protein (ASC), Interleukin-1 beta (IL1-β) and Absent In Melanoma 2 (AIM 2) were analysed with Taqman RT-PCR.

**Results:**

IL-1β cytokine expression was reduced by female sex hormones and notably the effect of estradiol was greatest in the preterm population. Female preterm neonates were more responsive to the anti-inflammatory effect of progesterone than male preterm infants. Term neonates had higher IL-1β, IL-18 and IL-1RA expression than preterm infants. Overall, in preterms, E2 and Pg lowered cytokine expression levels. Inflammasome gene expression profiles did not differ between preterm male and female neonates.

**Conclusion:**

Sex hormones altered the expression of multiple cytokines, and cytokine responses differ by sex. Gestation plays an important role in the inflammatory response, and we note term infants have a more robust profile while preterm infants are more responsive to hormonal stimulus. Female sex hormones have an important role in modulating neonatal immune response and may contribute to the female immune advantage.

**Impact:**

- Female sex hormones play an important role in modulating the neonatal immune response. This is reflected clinically by better bacterial clearance and improved sepsis outcome in females.
- This study aims to test the hypothesis that male and female neonates differ in their cytokine and inflammasome response and in response to endotoxin and sex steroid hormones. In preterm infants there is a sex difference in IL-1b responses which is observed rapidly following endotoxin stimulation.
- Differing immune responses according to sex has implications for future clinical application. Further work to characterise these sex differences may help in guiding therapy during sepsis.

## Introduction

Neonates are at increased risk of sepsis compared to adults and older children which is especially marked in the preterm population.^1,2^ While there are many potential reasons for this increased risk, the altered state of the preterm immune system is a significant contributor.^3^ The incidence of neonatal sepsis is estimated to be approximately 22 per 1000 live births with an estimated case fatality rate of between 11–19%.^4^ Premature neonates have deficits in almost all areas of innate and adaptive immunity^5,6^ and it is well established that male sex is a risk factor for neonatal sepsis.^7,8^ Cytokines have important roles in cell signalling and have a diverse range of biological functions. While preterm neonates with sepsis have the ability to mount pro- and anti-inflammatory cytokine responses,^9^ preterm neonatal immune cells have altered cytokine responses compared to term neonates and adults.^10,11^ The immune response of preterm neonates may be more weighted towards a pro- rather than anti-inflammatory response as reflected by the endotoxin response of macrophages in preterm neonates which have diminished IL-10 secretion following stimulation.^12^ Preterm infants are also more susceptible to infection however they can also experience a cytokine storm, characterized by elevated levels of pro-inflammatory cytokines. This systemic inflammation and its associated biochemical changes are linked to conditions such as necrotizing enterocolitis (NEC) and brain injury.^13–15^

The inflammasome is a multi-protein complex which results in both inflammatory cell death and the secretion of the markedly pro-inflammatory cytokines Interleukin-1β (IL-1β) and Interleukin-18 (IL-18).^16^ The NOD-, LRR- and pyrin domain-containing protein 3 (NLRP3) and absent in melanoma 2 (AIM2) inflammasomes are among the best studied and are critical components of innate immune response to infection.^17,18^ Both AIM2 and NLRP3 accumulate in the cytoplasm following activation and interact with Apoptosis-associated speck-like protein containing a CARD (ASC) to form an inflammasome complex resulting in the accumulation of pro-caspase-1, which then auto-cleaves. The resulting activated caspase 1 then undertakes the proteolytic conversion of the inactive forms of both IL-18 and IL-1β to their active forms. Inflammasome activation has been implicated in many disease processes^19–21^ though there is relatively little data in the preterm neonatal population. A study on placental inflammasome activity found that the NLRP3 inflammasome plays a role in the development of bronchopulmonary dysplasia (BPD), early sepsis, patent ductus arteriosus (PDA), and surgical NEC. The expression levels of NLRP3 varied in premature infants born between 24 to 29 weeks of gestation.^22^ In a study of premature infants, IL-1β produced by the inflammasome, was higher in infants who went on to develop late-onset sepsis.^23^ Recent work by De Biasi et al. demonstrated that plasma levels of IL-1β are similar in both preterm and term infants, but mRNA expression is lower in preterm infants. Additionally, they found that the inflammasome component NLRP3 decreases with LPS stimulation in preterm infants compared to term infants.^24^ However this study only included late preterm infants and none below 32 weeks gestation and in addition was performed on cord blood monocytes which is not equivalent to neonatal postnatal samples and LPS responses are suppressed in cord blood^25^ and therefore not comparable to neonatal samples.^26–28^

Immune responses differ between male and female preterm neonates. Male neonates have a poorer ability to balance the pro- and anti-inflammatory response leading to greater susceptibility to sepsis^7^ and sepsis-related inflammation.^8^ While cytokine responses to endotoxin may differ between sexes in neonates^29^ and adults,^30,31^ data on preterm infants is limited. Sex hormones, which increase with each trimester,^2^ are known to affect immune responses and may contribute to sex differences in immune responses after birth.^6^ Although clinical studies on the foetal immune response are limited, there is substantial scientific evidence indicating that hormones may play a significant role.^32,33^ Female sex hormones have been shown to modulate neonatal immune response with higher levels of steroid receptors than adults.^34^ Despite high levels of circulating female sex hormones in utero, little data is available on whether oestrogen (E2) and progesterone (Pg) affect the immune responses of male and female neonates differently.

We hypothesised that male and female neonates have differing cytokine responses and inflammasome gene expression which may impact clinical outcome. Our aim was to examine inflammasome and immune activation in response to inflammatory stimuli and sex steroid hormones in male and female term and preterm neonates.

## Methods

### Ethics and patient groups

This study was approved by the ethics committees of the Coombe Hospital, the National Maternity Hospital and the Rotunda Hospital, Dublin, Ireland.

#### Preterm infants

Premature neonates were eligible for inclusion if their birth weight was <1500 g and less than 32 weeks gestation. Premature neonates were not eligible for inclusion if there was a history of maternal substance abuse or confirmed serious congenital abnormalities (genetic, metabolic, cardiac), Table 1.

**Table 1:** Neonatal demographics of male and female preterm infants.

Parameter	Preterm males (*n* = 22)	Preterm females (*n* = 18)	*p* value
Gestation at delivery (weeks)	28.43 ( ± 1.793)	29.01 ( ± 2.241)	0.37
Gestation at sampling (weeks)	31.68 ( ± 2.461)	32.06 ( ± 2.636)	0.64
Birth weight (grams)	1058 ( ± 315.1)	1126 ( ± 296.6)	0.49
PROM, n (%)	5 (23)	2 (11)	0.43
Chorio, n (%)	0	1 (6)	0.46
NEC	9 (41)	3 (17)	0.17
Ventilation	9 (41)	3 (17)	0.17
PDA	2 (9)	3 (17)	0.64

Premature rupture of membranes (PROM), Choriamnionitis (Chorio), Necrotising enterocolitis (NEC), Patent ductus arteriosus (PDA), ventilation describes invasive ventilation required.

#### Term control infants

Peripheral blood was collected in healthy term controls recruited in the Coombe Hospital, Dublin within the first 48 h after delivery. Term controls were eligible for participation if they were having routine phlebotomy performed on the postnatal ward and did not have any clinical or laboratory evidence of infection or serious congenital abnormalities (genetic, metabolic, cardiac).

### Sampling

Whole blood from term (*n* = 26, 50% male) and preterm neonates (*n* = 40, 55% male) was collected in a sodium citrate anti-coagulated blood tube and analysed within two hours of phlebotomy. Preterm whole blood was collected from the first 72 h of birth. Whole blood was incubated at 37 °C for 1 h untreated (vehicle), with Lipopolysaccharide (LPS; E. coli 0111:B4: SIGMA Life Science, Wicklow, Ireland) at 10 ng/ml and or with Estradiol (10 nM) (SIGMA Life Science, Wicklow, Ireland), Progesterone (10 nM) (SIGMA Life Science, Wicklow, Ireland) or Pam3Cys-Ser-(Lys)4 trihydrochloride (Pam3CSK4) (TOCRIS bio-techne, Abingdon, UK) (5 ng/ml). After incubation the samples were centrifuged at 1500 rpm for 10 min at room temperature and serum stored at –80 °C for further analysis using multiplex ELISA.

### Cytokine evaluation

The following cytokines were analysed on a custom-made sandwich ELISA MULTI-SPOT assay plate from Meso Scale Diagnostics: Interleukin-18 (IL-18), Interleukin-1 receptor antagonist (IL-1RA), Interleukin-1 beta (IL-1β), and Interleukin-33 (IL-33). For analysis, serum of participants was transferred to 96-well MULTI-ARRAY plates and singlet analysis processed as per the manufacturer’s instructions (Meso Scale Diagnostics). The limits of detection were within expected ranges for each individual assay. The plates were analysed on a Meso Scale Diagnostics instruments. Results are presented in picograms/ml.^20,26^

### Qualitative RT-PCR

Whole blood in RNA later was thawed and RNA was extracted using the Ribopure blood kit (Thermo Fisher Scientific, Waltham, MA), following the manufacturer’s instructions. RNA purity and concentration were determined on the NanoDrop ND-100 Spectrophotometer and analysed using ND-1000 Ver.3.1.2 software. Total RNA, 1 µg, was reverse transcribed to single-stranded cDNA using the High-Capacity cDNA Archive Kit (Applied Biosystems) following the manufacturer’s protocol and stored at −80 °C until use. The settings for amplification were 10 min at 25 °C and 120 min at 37 °C and 5 min at 85 °C then hold at 4 °C. The evaluation of inflammasome gene expression was performed by TaqMan^®^ RT–PCR with probes for NLR family pyrin domain containing 3 (NLRP3) (Hs00918082_m1), apoptosis-associated speck-like protein (ASC) (Hs00203118_m1), Interleukin-1 beta (IL1-β) (Hs00174097_m1) and Absent In Melanoma 2 **(**AIM 2) (Hs00915710_m1). Human GAPD (GAPDH) Endogenous Control (NM_002046.3) was used for data normalization. The Quantstudio V5 was used for analysis, using the following conditions; 2 min at 50 °C, 10 min at 95 °C, 40 cycles of 95 °C for 15 s followed by 60 °C for 1 min and relative quantification (RQ) values with healthy term control calibrators were calculated using the 2^−ΔΔCt^ method.^35^

### Statistical analysis

Data analysis was undertaken using IBM SPSS 24.0 and Microsoft Excel. Graphical comparisons were made using GraphPad Prism 8.0. Categorical variables were compared using x2 test. Unpaired, continuous data were compared using the independent sample *t* test if parametric or the Mann–Whitney U test if non-parametric. Paired, continuous data were compared using paired *t* tests if parametric or Wilcoxen matched pair analysis if non-parametric. Statistical significance was set at *p* < 0.05 for all analyses.

## Results

### Patient characteristics

Seventy-two newborn infants were enroled including 40 preterm neonates (18 female, 22 male) with a mean gestation at birth (mean ± SD) of 28.69 ± 2 weeks and corrected gestation at the time of sampling was 31.85 ± 2.5 weeks. The mean birth weight of preterm neonates was 1089 ± 304.9 g. Gestation at birth, corrected gestation at sampling and birth weight did not differ significantly between male and female preterm neonates. There were no significant differences between the incidence of premature rupture of membranes, necrotising enterocolitis, chorioamnionitis, patent ductus arteriosus and ventilation status between males and females (Table 1). None of the infants had culture proven sepsis prior to sampling. The ethics committee allowed gestation and sex only to be collected on controls who were then anonymised but equal numbers of healthy males and females were recruited within the first week of life.

#### Effect of endotoxin on cytokine levels in preterm versus term infants

**Fig. 1:**
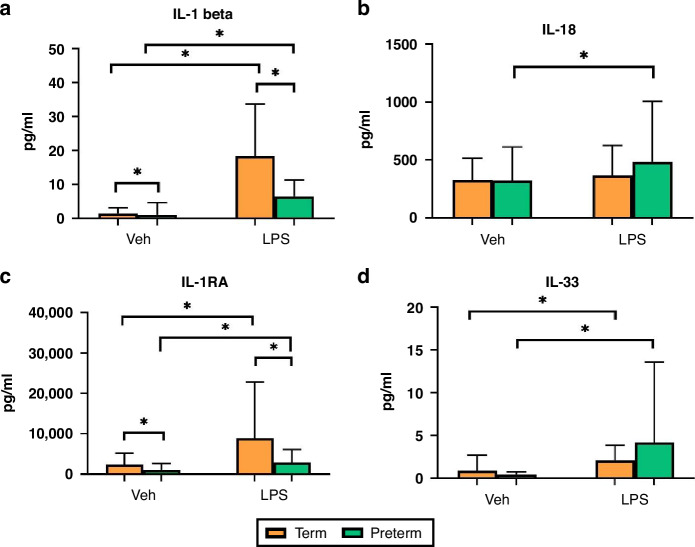
Enotoxin cytokine responses in preterm vs. term neonates. Serum IL-1β (**a**), IL-18 (**b**), IL-1RA (**c**) and IL-33 (**d**) levels in term (*n* = 32) and preterm (*n* = 40) neonates at baseline versus LPS stimulation. Values displayed represent mean ± standard deviation of pg/ml. *=significant at *p* < 0.05.

IL-1β, IL-1RA and IL-33 increased significantly in both term (*p* < 0.0001, *p* = 0.017, *p* = 0.020 respectively) and preterm (*p* < 0.0001, *p* = 0.002, *p* = 0.014 respectively) neonates following LPS treatment (Fig. 1a, c, d). IL-1β and IL-1RA were higher in term neonates both at baseline (*p* = 0.0002, *p* = 0.017, respectively) and following LPS treatment (*p* < 0.0001, *p* = 0.017) compared to preterm neonates (Fig. 1a, c). IL-18 increased significantly in preterm neonates following LPS treatment (*p* = 0.0001) but not in term neonates (Fig. 1b).

#### Sex and cytokine responses in preterm versus term infants

**Fig. 2:**
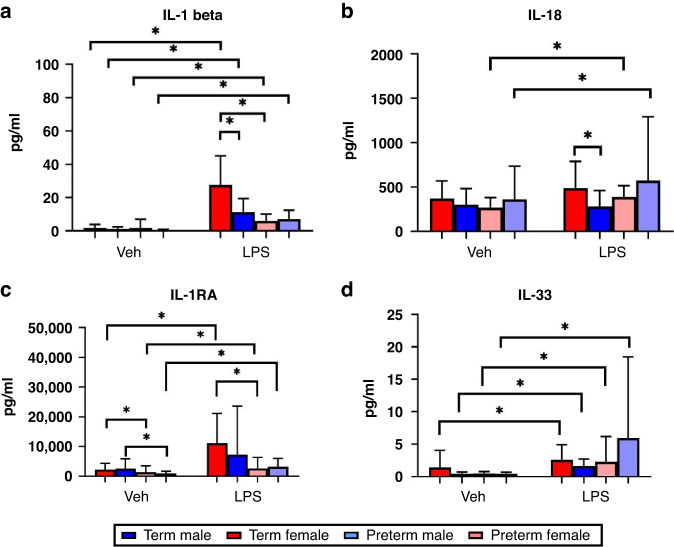
Sex differences in cytokine responses. Serum IL-1β (**a**), IL-18 (**b**), IL-1RA (**c**) and IL-33 (**d**) levels in male (*n* = 16) and female (*n* = 16) term and preterm (*n* = 22 male and *n* = 18 female) neonates at baseline versus LPS stimulation. Values displayed represent mean ± standard deviation of pg/ml. *=significant at *p* < 0.05.

IL-1β increased significantly in term male (*p* < 0.0001), term female (*p* < 0.0001), preterm male (*p* < 0.0001) and preterm female (*p* = 0.016) neonates following LPS treatment. IL-1β was higher in term female neonates compared to both term male (*p* = 0.002) and preterm female (*p* < 0.0001) neonates following LPS treatment (Fig. 2a). IL-18 increased significantly in both preterm male (*p* = 0.006) and preterm female (*p* = 0.008) neonates following LPS treatment compared to term neonates of the same sex. IL-18 was significantly higher in term females following LPS treatment compared to term males (*p* = 0.024) (Fig. 2b).

IL-1RA increased significantly in term female (*p* = 0.005), preterm male (*p* = 0.0007) and preterm female (*p* = 0.004) neonates following LPS treatment. IL-1RA was significantly higher in term female neonates at baseline (*p* = 0.025) and following LPS treatment (*p* = 0.004) compared to preterm female neonates. IL-1RA was significantly higher in term male neonates at baseline compared to preterm male neonates (*p* = 0.041) (Fig. 2c). IL-33 increased significantly in term male (*p* = 0.0005), term female (*p* = 0.005), preterm male (*p* = 0.044) and preterm female neonates (*p* < 0.0001) following LPS treatment (Fig. 2c).

#### The effect of oestrogen on endotoxin-treated cytokine levels in preterm versus term infants

**Fig. 3:**
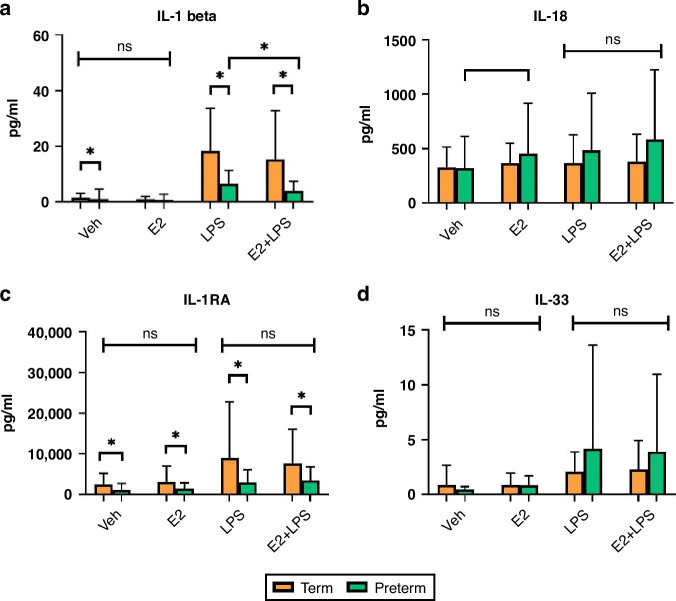
Estradiol modulates neonatal cytokine reponses. Serum IL-1β (**a**), IL-18 (**b**), IL-1RA (**c**) and IL-33 (**d**) levels in E2-treated term (*n* = 32) and preterm (*n* = 40) neonates compared with baseline and LPS treated samples. Values displayed represent mean ± standard deviation of pg/ml. *=significant at *p* < 0.05.

IL-1β was higher in term neonates at baseline (*p* = 0.0002), in samples treated with LPS (*p* < 0.0001) and E2 + LPS (*p* = 0.004) compared to preterm neonates. IL-1β decreased significantly in preterm neonatal LPS-treated samples when incubated concurrently with E2 (*p* = 0.049) (Fig. 3a). Baseline IL-18 increased significantly in preterm neonates following E2 treatment (*p* = 0.016) (Fig. 3b).

IL-1RA was higher in term neonates compared to preterm neonates at baseline (*p* = 0.017) and following treatment with E2 (*p* = 0.037), LPS (*p* = 0.017) and E2 + LPS (*p* = 0.028). Treatment with E2 did not affect IL-1RA levels (Fig. 3c). Treatment with E2 did not affect IL-33 levels (Fig. 3d).

#### The effect of oestrogen on endotoxin-treated cytokine levels in term and preterm infants divided by sex

**Fig. 4:**
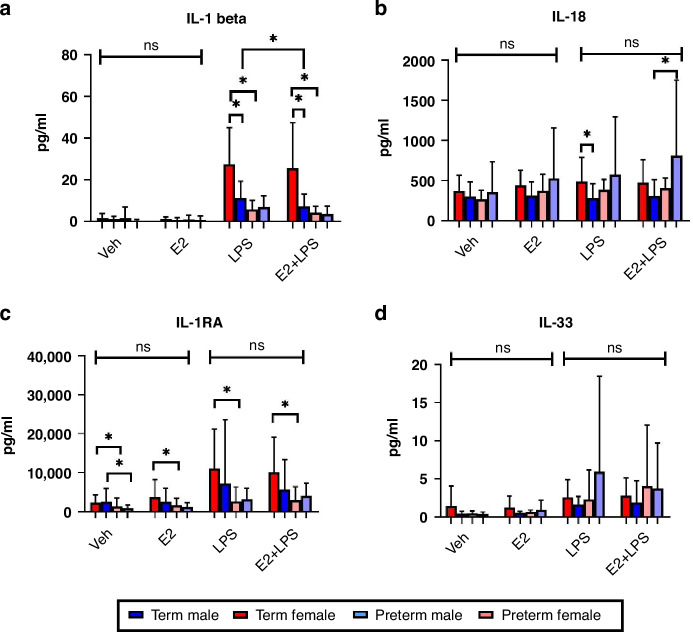
Sexual dimorphism in E2 treated cytokine responses Serum IL-1β (**a**), IL-18 (**b**), IL-1RA (**c**) and IL-33 (**d**) levels in E2-treated male (*n* = 16) and female (*n* = 16) term and preterm (*n* = 22 male and *n* = 18 female) neonates compared with baseline and LPS treated samples. Values displayed represent mean ± standard deviation of pg/ml. *=significant at *p* < 0.05; ns-non-significant.

Term females had higher IL-1β than both term male (*p* = 0.002) and preterm female (*p* < 0.0001) neonates following LPS treatment. Term females had higher IL-1β than both term male (*p* = 0.002) and preterm female (*p* = 0.002) neonates following E2 + LPS treatment. IL-1β was significantly lower in LPS-treated samples when incubated with E2 in term male neonates only (*p* = 0.043) (Fig. 4a). IL-18 was significantly higher in term females following LPS treatment compared to term males (*p* = 0.024). IL-18 was significantly higher in preterm males following E2 + LPS treatment compared to term male neonates (*p* = 0.040). IL-18 levels were not significantly affected by treatment with E2 (Fig. 4b).

Term female neonates had higher IL-1RA than preterm female neonates at baseline (*p* = 0.025) and following treatment with E2 (*p* = 0.037), LPS (*p* = 0.004) and E2 + LPS (*p* = 0.012). Term male neonates had higher IL-1RA than preterm male neonates at baseline (*p* = 0.041) (Fig. 4c). IL-33 levels were not significantly affected by treatment with E2 (Fig. 4d).

#### The effect of combined oestrogen and progesterone on endotoxin-treated cytokine levels

**Fig. 5:**
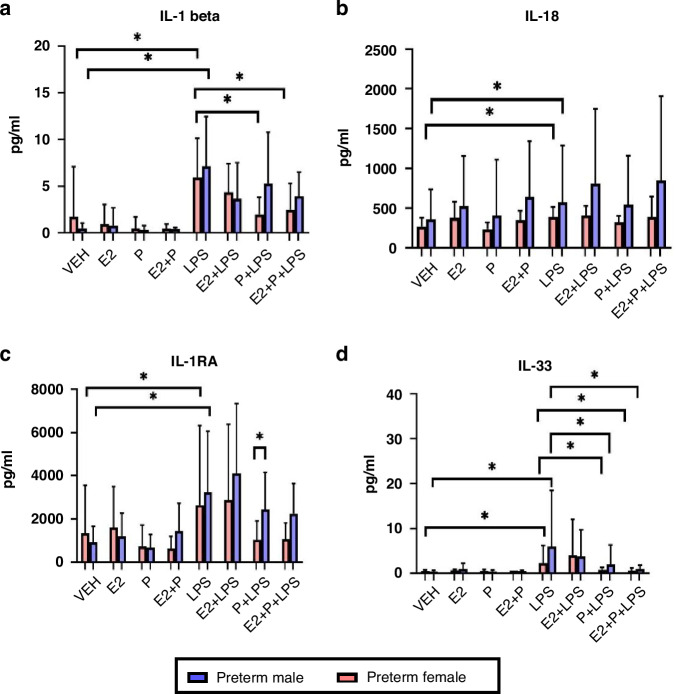
Hormonal effects of E2 and Pg in preterm neonates. Serum IL-1β (**a**), IL-18 (**b**), IL-1RA (**c**) and IL-33 (**d**) levels in hormone-treated male (*n* = 22) and female preterm neonates (*n* = 18). Progesterone combined with estradiol stimulus is compared with baseline and LPS values. Values displayed represent mean ± standard deviation of pg/ml. *=significant at *p* < 0.05.

To assess the potential role of sex hormones in immune modulation, cytokine levels in preterm neonatal samples treated with E2 and Pg for 1 h either alone or in combination with endotoxin were measured. IL-1β increased significantly in both male (*p* < 0.0001) and female (*p* = 0.016) preterm neonates following LPS treatment. IL-1β was significantly lower in LPS-treated samples when incubated with Pg (*p* = 0.007) or E2+Pg (*p* = 0.047) in female preterm neonates only (Fig. 5a). IL-18 increased significantly in both preterm male (*p* = 0.006) and female (*p* = 0.008) neonates following LPS treatment (Fig. 5b).

IL-1RA increased significantly in male (*p* = 0.0007) and female (*p* = 0.004) preterm neonates following LPS treatment. IL-1RA was significantly higher in preterm male neonates when treated with Pg+LPS compared to preterm female neonates (*p* = 0.027) (Fig. 5c). IL-33 increased significantly in both preterm male and preterm female neonates following LPS treatment. IL-33 was significantly lower in both male (*p* = 0.001) and female preterm (*p* = 0.005) LPS-treated samples when incubated with Pg. IL-33 was significantly lower in both male (*p* = 0.049) and female (*p* = 0.003) preterm LPS-treated samples when incubated with E2+Pg (Fig. 5d).

#### The effect of combined oestrogen and progesterone on Pam3CSK4-treated cytokine levels

**Fig. 6:**
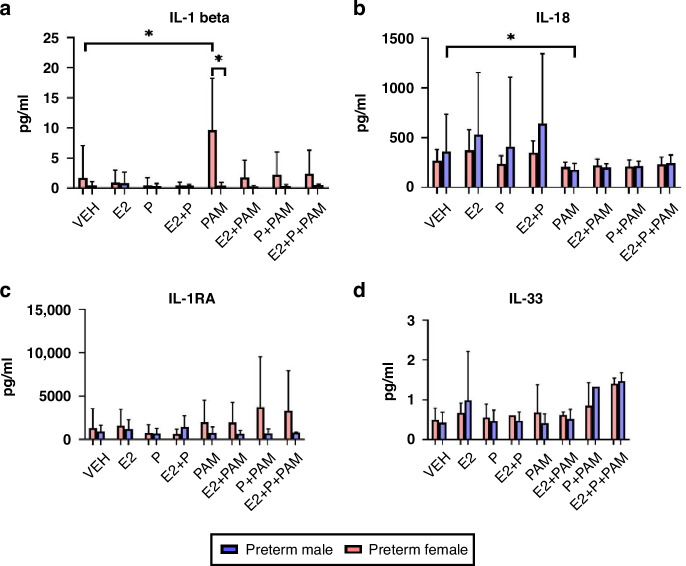
Pam3CSK4 modulates the preterm female immune response. Serum IL-1β (**a**), IL-18 (**b**), IL-1RA (**c**) and IL-33 (**d**) levels in pam3CSK4-treated male (*n* = 22) and female (*n* = 18) preterm neonates. Values displayed represent mean + standard deviation of pg/ml. *=significant at *p* < 0.05.

Gram positive organisms such as CONS are among the commonest and most important causes of sepsis in the preterm population^36^ and TLR2 is important in the recognition of such gram-positive bacteria. To assess the potential role of sex hormones in immune modulation, cytokine levels in samples treated with E2 and Pg for 1 h either alone or in combination with the TLR2 agonist Pam3CSK4 were measured. IL-1β increased significantly in preterm female neonates treated with Pam3CSK4 (*p* = 0.027) but not preterm males to their male counterparts (*p* = 0.006) IL-18 decreased significantly in preterm males but not females following Pam3SCK4 treatment (p = 0.018). IL-1RA and IL-33 did not differ between groups with the addition of Pam3CSK4 (Fig. 6a–d, respectively). The combination of E2 and or Pg did not change the expression levels within the preterms or between the sexes.

#### The effect of sex on inflammasome gene expression

Given the sex differences in IL-1β and IL-18 observed, inflammasome gene expression was then examined in term and preterm neonates. RNA was extracted from whole blood, cDNA synthesisedand qRT-PCR was undertaken to examine the expression of NLRP3, AIM2, ASC and IL-1β. Fold change was calculated relative to healthy term control neonates. Fold change expression did not differ between term and preterm neonates for NLRP3, AIM2, ASC and IL-1β (Supplementary Fig. S1). When results were examined separately by sex, results did not differ between groups (Supplementary Fig. S2).

## Discussion

Differences in sepsis outcomes between boys and girls remain poorly understood, with studies indicating that sepsis is more prevalent in males than females, showing an annual relative risk of 1.3 times that of females. Additionally, among septic shock patients admitted to ICUs, males are more prevalent than females.^37^ This study found significant changes in the inflammasome-related cytokines IL-18 and IL-1β between males and females, although no differences in gene expression between sexes were observed. Steroid hormones have the potential to regulate cytokine expression.

We found that E2 and Pg had potentially important effects on the expression of the cytokines studied, supporting the suggestion that sex hormones may have an important role in preterm male and female neonatal immune responses.^6^ While IL-1RA did not change significantly with the addition of E2 it is notable that IL-1RA was higher in preterm males in samples treated with Pg. IL-1β however, was affected by female sex hormones and notably the effect of E2 was greatest in the preterm population and female preterm neonates appeared more responsive to the anti-inflammatory effect of Pg.

Both term and preterm neonates showed significant elevations in IL-1β following LPS treatment, with term neonates exhibiting greater expression at baseline and in LPS-treated samples. Supported this finding, Strunk et al. have previously described an increase in IL-1β expression by preterm mononuclear cells in a gestationally-dependent manner.^38^ Term female neonates had the most robust response in endotoxin-treated samples, reflecting their gestational and sex-related immune advantage and supported by Bessler et al. ^10,39^ Female preterm neonates showed significant elevation in IL-1β following Pam3CSK4 treatment in contrast to preterm male neonates. This differential response may contribute to the heightened susceptibility to gram-positive infections observed in male neonates. Kim-Fine et al. found greater IL-1β in male neonatal cord blood in response to LPS stimulation.^29^ Clinical studies have implicated IL-1β in chronic lung disease^40^ and sepsis-related brain injury in neonates,^41,42^ highlighting its role in preterm clinical outcomes. IL-1β decreased significantly in preterm samples treated with endotoxin and E2, particularly in term male neonates. Preterm female neonates were more sensitive to the immunomodulation effect of Pg, showing significant reductions in IL-1β between LPS-treated samples and those treated with LPS and Pg combined.

IL-18 expression was similar at baseline in term and preterm neonates, but only preterm neonates showed significant increases following LPS treatment. Zasada et al. found higher IL-18 in extremely preterm neonates compared to older gestation.^23^ The greater increase in IL-18 in preterm neonates following endotoxin stimulation may reflect the immaturity of preterm innate immunity. IL-18 levels decreased significantly in preterm male neonates treated with Pam3CSK4, but not in females. Female sex hormones did not affect IL-18 levels in term or preterm neonates. The aetiology of sex differences in IL-1β and IL-18 may involve differences in cellular components, including pro-cytokine production, caspase activity, or TLR-mediated inflammasome priming.

While E2 did not significantly affect the expression of IL-33, preterm male and female LPS-treated samples had a significant reduction in IL-33 when concurrently incubated with Pg either alone or in combination with E2. Giannoni et al. found that E2 and Pg strongly inhibit several important aspects of innate immune responses in cord blood mononuclear cells from healthy term newborns exposed to LPS, including TNF and IL-6, irrespective of sex.^34^ Various agents including vitamin A,^43^ steroids^44^ and NSAIDs^45^ have been suggested as potential modulators of neonatal cytokine response and we have identified a similar and potentially important role for female sex hormones in moderating neonatal cytokine responses. Both E2 and Pg showed potential to alter innate immune responses in term and preterm neonates and notably these responses often differed depending on neonatal gestation and sex. Trotter et al. reported improved postnatal bone mineral accretion and less chronic lung disease with estradiol and progesterone replacement therapy in female infants with a median gestational age of 26.6 weeks with normal neurodevelopmental follow up in the treated group and a trend towards delayed development in the control group at 15 months.^46,47^ There was also a time-dependant reduced risk of cerebral palsy, spasticity, and ametropia 5 yrs post treatment.^48^ There is a potential role for immunomodulation in the treatment and prevention of neonatal sepsis and sepsis-related neuroinflammation in neonates. The effects we have noted are important in the context of antenatal steroids and maternal progesterone, two medications often administered to women with threatened preterm birth.

The differences in responses that we have identified between the sexes has important implications in the future interpretation of these studies and clinical application. The inflammasome genes NLRP3, AIM2, ASC and IL-1β showed no difference in fold change gene expression between term and preterm neonates of either sex. No other studies could be identified which compared inflammasome gene expression between the sexes in neonates. Sharma et al. explored immaturity of the inflammasome in cord blood using western blot in premature neonates and found that ASC levels were similar to those of adults, though NLRP3 protein expression was reduced upon stimulation with endotoxin.^49^ This may suggest that the inflammasome components studied may not vary between term and preterm neonates in the unstimulated state and that immaturity of the preterm inflammasome may only become evident following immune stimulation. Our group have published works on term infants with neonatal encephalopathy (NE) and healthy controls and found the NLRP3 inflammasome dysregulated in the NE infants with corresponding downstream cytokine responses.^20,21^

Few studies have evaluated the impact of sex on neonatal blood cytokine responses, often using cord blood^29^ or amniotic fluid.^50^ This study is the first to evaluate cytokines in samples acquired postnatally in term and preterm neonates using sex as a key differentiator. We have shown that cytokine responses are distinct in term and preterm neonates and differ between males and females. Term female neonates have the most robust response with higher levels of both pro- and anti-inflammatory cytokines. This balance in pro- and anti-inflammatory responses is reflected clinically by better bacterial clearance and improved sepsis outcome. Female sex hormones have an important role in modulating neonatal immune response and have a role in the female immune advantage.

Additionally, testosterone levels in male neonates can enhance immune cell activity and cytokine production, modulate inflammatory responses to reduce harmful inflammation, and indirectly support a stronger immune system.^51^ However, these findings may be influenced by the specific conditions under which the studies were conducted, including the age, health status, and environmental factors affecting the neonates. This study did not evaluate testosterone levels or their impact on neonatal immune responses. The focus was solely on the pro- and anti-inflammatory cytokine responses in term and preterm neonates, with sex as a key differentiator.

A limitation of this study is the small sample size, comprising 18 female and 22 male preterm infants. Although a total of 40 preterm infants is relatively large for a study involving neonatal peripheral whole blood, this limits the statistical power, particularly when analysing results by neonatal sex. Consequently, important differences within the cohort may have been overlooked. While cytokines hold significant promise as biomarkers in neonatal sepsis, we have identified notable sex differences in their circulating levels. Previous studies have demonstrated that cytokine profiles of preterm neonates with early-onset sepsis (EOS) and late-onset sepsis (LOS) are distinct,^52^ as are those of extremely low birth weight (ELBW) neonates with various forms of microbiological sepsis,^53^ further complicating their application. It has been suggested that circulating cytokine levels may not increase linearly with gestational age,^54^ and antenatal inflammation can potentially alter cytokine levels in preterm neonates.^55^ These factors may contribute to the apparent discrepancies in the literature and should be considered in future research.

Our results highlight the immaturity of preterm neonatal cytokine responses and possible sex differences in cytokine expression. E2 and Pg may modulate neonatal immune response early in life. Term female neonates had the highest levels of pro- and anti-inflammatory cytokines following stimulation, reflecting their ability to fight infection while avoiding excessive inflammation. Elevated cytokine responses in term female neonates compared to preterm females may reflect the advantage of increased gestation, while their improved responses compared to male term neonates may represent the effect of sex. There is a need to consider sex when utilising steroids both in terms of dose and timing of these medications and that there are sex-specific effects of these therapeutic interventions.

## Supplementary information

Supplementary Figures

## Data Availability

The datasets generated during and/or analysed during the current study are available from the corresponding author on reasonable request.
