# Supplementary material for: Sex differences in preterm cytokine and inflammasome responses and modulation by exogenous sex steroids
Source: Pediatr Res. 2025 Sep 24;99(4):1621–9. doi: 10.1038/s41390-025-04350-0 (PMC13102689; doi:10.1038/s41390-025-04350-0)
Supplement: Supplementary file 1 — Supplementary Figures [file 41390_2025_4350_MOESM1_ESM.pdf]

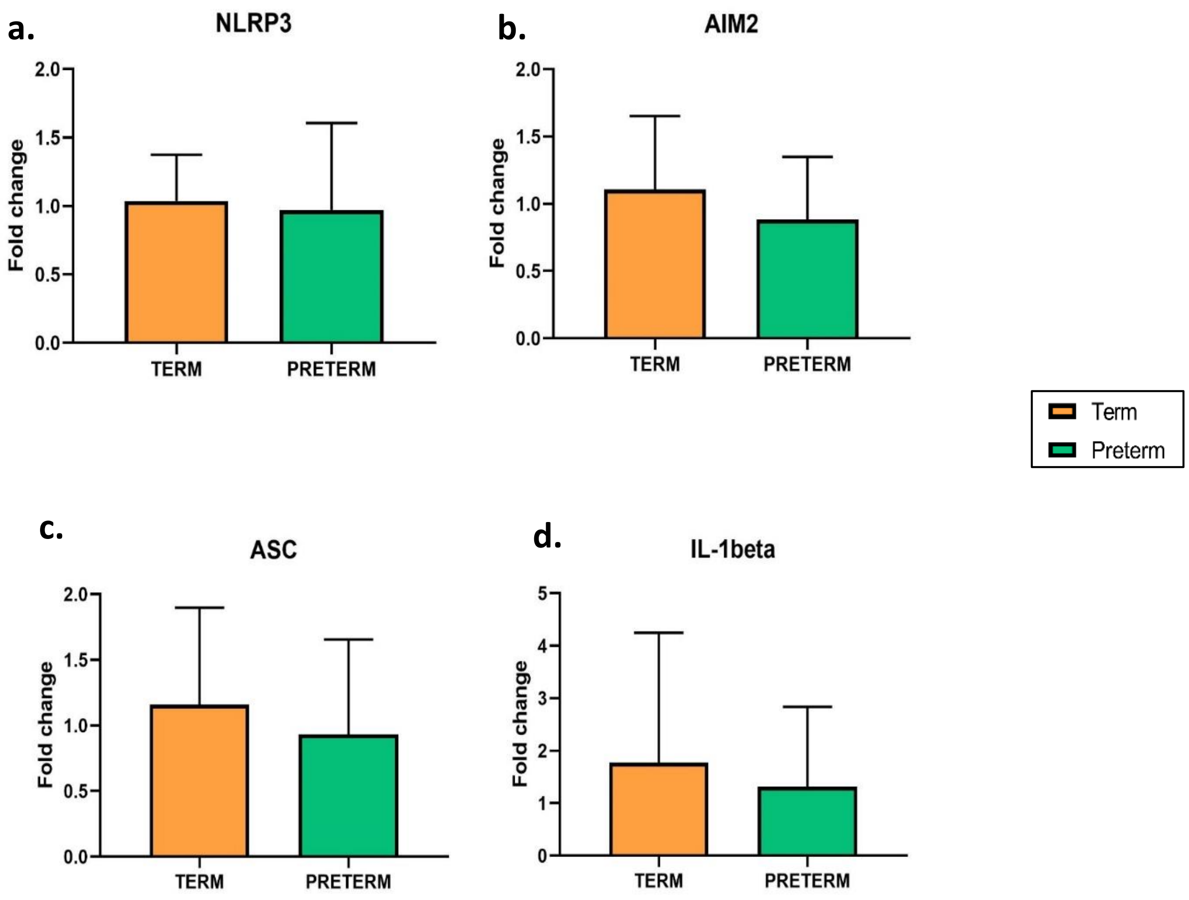

Supplemental Figure S1: Inflammasome gene expression in term and preterm neonates.

Inflammasome gene expression in term (n=9) and preterm (n=10) neonates. Values displayed represent mean ± standard deviation fold change expression. Values did not differ between groups. a) NLRP3; b) AIM2; c) ASC; d) IL-1β.

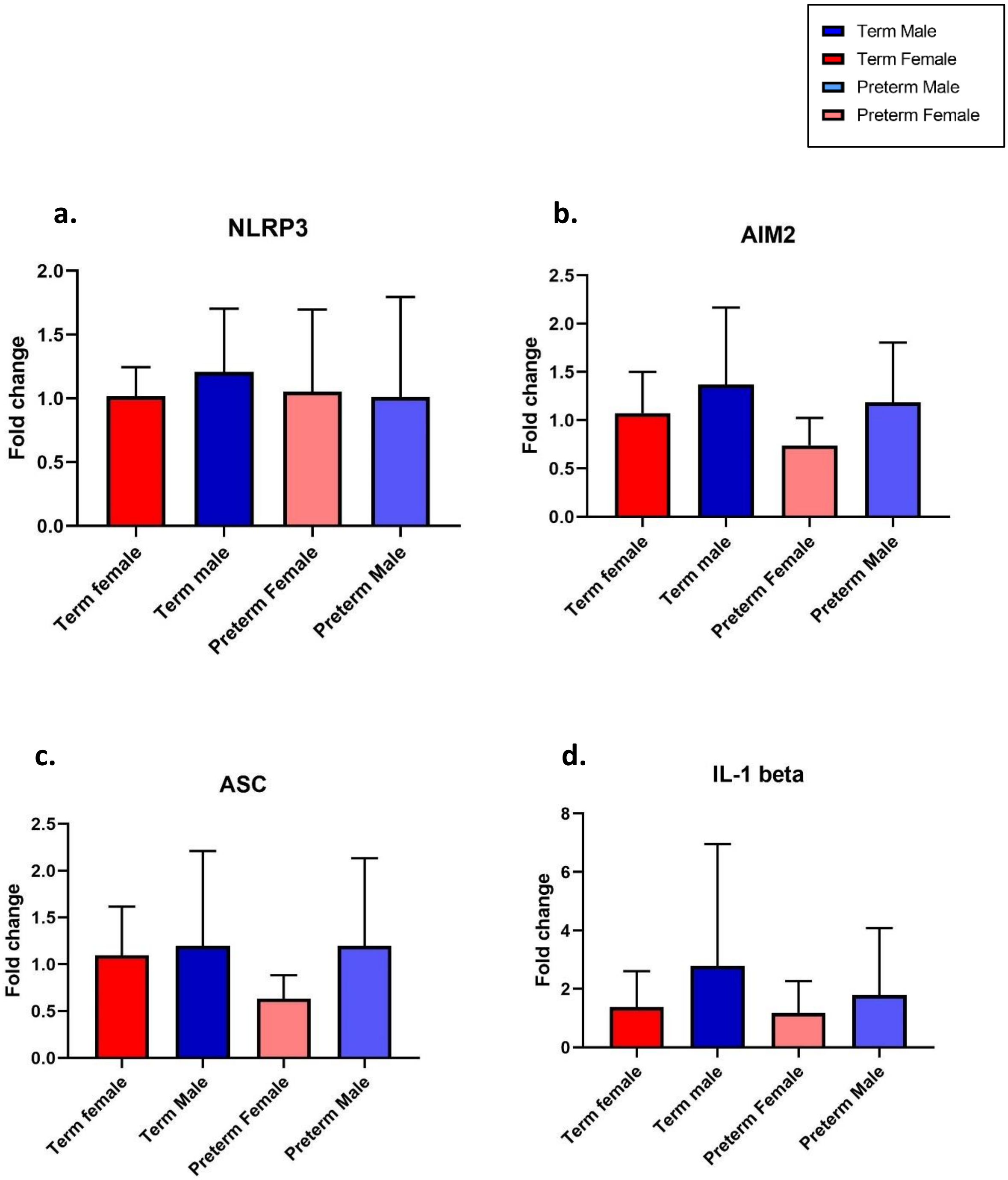

Supplemental Figure S2: Inflammasome gene expression in male and female term and preterm neonates.

Inflammasome gene expression in male (n=5) and female (n=4) term and preterm (n=5 male and n=5 female) neonates. Values displayed represent mean ± standard deviation fold change expression. Values did not differ between groups. a) NLRP3; b) AIM2; c) ASC; d) IL-1β.
